# A notice of correction

**DOI:** 10.3325/cmj.2012.53.513

**Published:** 2012-10

It has come to our attention that a part of an article published in the *Croatian Medical Journal* (Auer J, Porodko M, Berent R, Punzengruber C, Weber T, Lamm G, Eber B. Transient left ventricular apical ballooning mimicking acute coronary syndrome in four patients from central Europe. Croat Med J. 2005;46(6):942-9) was previously published as a letter to the editor in the *International Journal of Cardiology* (Auer J, Porodko M, Berent R, Punzengruber C, Weber T, Lamm G, Eber B. Left ventricular apical ballooning – a novel cardiac disease mimicking acute coronary syndrome: a case report in a Caucasian patient. Int J Cardiol. 2006;106(3):398-400, published online March 22, 2005). The letter published in the *International Journal of Cardiology* includes the description of the first case described in the article published in the *Croatian Medical Journal*, while the other cases presented in the *Croatian Medical Journal* are original. The authors have apologized.

